# Surface Characteristics of Milled and 3D Printed Denture Base Materials Following Polishing and Coating: An In-Vitro Study

**DOI:** 10.3390/ma13153305

**Published:** 2020-07-24

**Authors:** Pablo Kraemer Fernandez, Alexey Unkovskiy, Viola Benkendorff, Andrea Klink, Sebastian Spintzyk

**Affiliations:** 1 Department of Prosthodontics, University Hospital Tuebingen, Osianderstrasse 2-8, 72076 Tübingen, Germany; pablo.kraemer-fernandez@med.uni-tuebingen.de (P.K.F.); viola.benkendorff@med.uni-tuebingen.de (V.B.); andrea.klink@med.uni-tuebingen.de (A.K.); 2 Department of Prothodontics, Geriartric Dentistry and Craniomandibular Disorders, Charité-Universitätsmedizin Berlin, Corporate Member of Freie Universität Berlin, Humboldt-Universität zu Berlin and Berlin Institute of Health, Aßmannshauser Str. 4-6, 14197 Berlin, Germany; 3 Department of Dental Surgery, Sechenov First Moscow State Medical University, Trubetskaya Str. 8-2, 119991 Moscow, Russia; 4 Section Medical Materials Science and Technology, University Hospital Tuebingen, Osianderstrasse 2-8, 72076 Tuebingen, Germany; sebastian.spintzyk@med.uni-tuebingen.de

**Keywords:** additive manufacturing, digital denture, full denture, edentualism, surface roughness, gloss, rapid manufacturing

## Abstract

(1) Background: To date, no information on the polishability of milled and 3D-printed complete denture bases has been provided, which is relevant in terms of plaque accumulation. (2) Methods: three groups (*n* = 30) were manufactured using the cold-polymerization polymethilmethacrilate, milling (SM) and 3D printing (AM). 10 specimens of each group were left untreated (reference). 10 more specimens were pre-polished (intermediate polishing) and 10 final specimens were highgloss polished. An additional 20 specimens were 3D printed and coated with the liquid resin (coated), 10 of which were additionally polished (coated + polished). For each group R_a_ and R_z_ values, gloss value and REM images were obtained. (3). The “highgloss-polished” specimens showed statistically lower R_a_ and R_z_ values in the SM, followed by AM and conventional groups. In the AM group statistically lower surfaces roughness was revealed for highgloss-polished, “coated + polished”, and “coated” specimens, respectively. (4) Conclusions: The milled specimens demonstrated superiors surface characteristics than 3D printed and conventionally produced after polishing. The polished specimens demonstrated superior surface characteristics over coated specimens. However, the surface roughness by both polished and coated specimens was within the clinically relevant threshold of 0.2 µm.

## 1. Introduction

For a successful rehabilitation of edentulous patients, the commonly used prosthetic appliances must meet certain qualitative criteria, including a smooth resin surface [1]. Abrasive wear during mastication and mechanical cleaning, poor dental hygiene and decreased salivation may lead to a high plaque accumulation and cause stomatitis and oral candidiasis [2]. For this reason, either mechanical or chemical polishing, and also coating procedures, are usually conducted to decrease the microbial adherence of denture surface [1,3,4]. A hydrophobic surface with a high surface energy and roughness is potentially more vulnerable to plaque accumulation [5,6,7,8]. According to in-vitro studies, a surface roughness (R_a_) of 0.2 µm was set as the clinically acceptable value [9,10].

In the last decade, due to a wide integration of computer aided design (CAD) and computer aided manufacturing (CAM) in dentistry, an increasingly large number of novel technical and clinical protocols have been introduced for complete denture manufacturing in a digital workflow [11,12,13]. In the pioneer digital technical approaches the denture bases and teeth have been produced in subtractive way using milling machines [14]. A wide variety of pre-polymerized polymethyl methacrylate (PMMA) blocks have been introduced on the market and controversial data was provided on surface roughness values. Thus, either comparable or a decreased surface roughness in comparison to heat and auto-polymerized denture resin was observed for milled denture bases [15,16]. In contrast, Srinivasan et al. reported greater R_a_ values for milled PMMA [17]. Nevertheless, subtractively produced denture bases showed reduced candida adherence compared to conventionally produced prosthesis bases [18,19].

In recent times, additive manufacturing of denture teeth and bases was reported using 3D printing technologies [20,21]. With printable materials available on the market, it has also become possible to produce well-fitting dentures using additive technologies. The positive patient reported outcomes were revealed for this method of edentualism rehabilitation in a digital workflow [22].

As an alternative to conventional polishing, utilization of coating materials for additively-produced denture bases may be opted [23].

According to the topical literature, no evidence is provided, which among subtractive and additive manufacturing approaches may yield a smoother denture surface after mechanical polishing. Furthermore, the coating procedure must be critically evaluated towards the conventional polishing, while considering also the working time quantification. These aspects may be relevant in terms of bacterial adhesion, long-term success and efficiency of the whole rehabilitation. Thus, the aim of the present study was to investigate the polishability of milled and 3D printed denture materials and evaluate the effect of coating on the denture surface roughness. The first null hypothesis of this study was that there would be no significant differences in surface roughness between polished conventional, subtractive and additive denture materials; the second null hypothesis is that the coated additive specimens would provide at least the same surface roughness as the polished ones.

## 2. Experimental Section

### 2.1. Specimens Manufacturing

**Figure 1:**
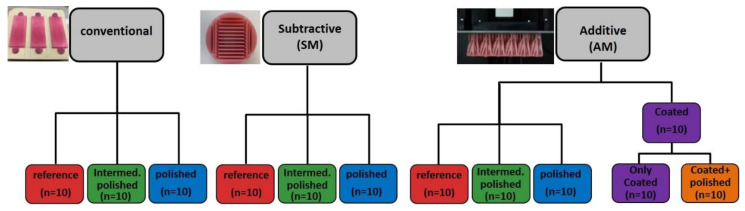
Study flowchart. Three different manufacturing approaches with various surface treatment protocols.

For analysis of polishability, three groups of specimens were fabricated, imitating the different manufacturing protocols for complete denture production: conventional (Con), subtractive (SM), and additive (AM) (Figure 1). The specimens of 2 × 2.5 × 5 cm in width, height and length were selected to imitate the palatal surface of a full denture prosthesis.

#### 2.1.1. Conventional

**Figure 2:**
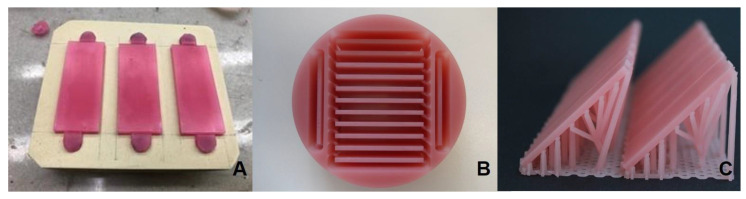
(**A**): Conventional specimens constructed in wax for further casting process. (**B**): Subtractively manufactured specimens in the polymethyl methacrylate (PMMA) block. (**C**): Additively manufactured specimens with the 45° printing orientation.

For production of the conventional group, the specimens of aforementioned dimensions were sculpted manually from wax (Modellierwachsplatten-Rosa Spezial, Omnident Dental-Handelsgesellschaft mbH, Rodgau Nieder-Roden, Germany) and prepolished using SitoPadSchwamm (Sito International GmbH & Co. KG, Hergatz, Germany). The wax specimens were embedded into class IV stone (YellowStar Klasse 4 Dental GmbH, Augsburg, Germany) to produce a two-part stone mold (Figure 2A). Both stone mold parts were isolated from each other (Isofix 2000, Renfert GmbH, Hilzingen; Germany). After the stone was set, the wax specimens were boiled out from the negative mold. The stone mold was left in water for 20 min and afterwards isolated against resin with Aislar PALA (Kulzer GmbH, Hanau, Germany). The auto-polymerizing resin (Aesthetic Blue, Candulor AG; 8152 Glattpark (Opfikon), Switzerland) was mixed according to the manufacturers’ specifications and applied into the mold. It was left in the pressure pot (Polymax 1, Dreve, Unna, Germany) for 20 min under 45 C and 2,5 bar. Afterwards, the polymerized specimens were extracted from the stone mold and stored in the black container till all other specimens were manufactured.

#### 2.1.2. Subtractive

A specimen of mentioned dimensions was designed virtually in CAD software (Fusion360, V. 2.0.5688, Autodesk). The stereolithography (STL) file was exported into the AM-software (DentalCAM, V.7.07.00.P3, vhf camfacture AG, Ammberuch, Germany) and positioned within the block of pre-polymerized PMMA (Vita Vionic Base Deep-Pink, Vita, Bad Säckingen, Germany). The specimens (*n* = 10) were milled wet (VHF S1, vhf camfacture AG, Ammberuch, Germany) (Figure 2B).

#### 2.1.3. Additive

The same virtual design in STL format was uploaded in the slicing software (Netfabb Premium 2019, Rapidshape Engine Ver. 2020.1.881.341, Autodesk, California, USA). The specimens were oriented 45º to the printing table. The supporting structures were applied in the nesting software. The specimens were printed at 50 µm layer thickness with Rapidshape D30II (Rapid Shape GmbH, Heimsheim, Deutschland), a direct light processing (DLP) 3D Printer with wavelength of 385 nm, printed from aliphatic urethane dimethacrylate material (Freeprint denture, Detax, Ettlingen, Germany) (Figure 2C). Afterwards, the printed specimens were put in an ultrasonic bath with isopropanol (99%) twice for 2 min each and then polymerized in the light curing unit (Otoflash G171, NK optics, Baierbrunn, Germany) with nitrogen gas with two times 2000 flashes with a 5 min break in between.

### 2.2. Polishing

All manufactured specimens were polished with only one operator in a clinical setting, in order to ensure approximately the same pressure of the polishing tools on the specimens. For each manufacturing method, 10 specimens were left untreated, constituting thereby the “reference” group (*n* = 10). 10 more specimens of each production method were roughly polished using only grinding paper, constituting the “intermediate-polish” group (*n* = 10). 10 more specimens were polished till the operator evaluated optically a clinical acceptable gloss shine, constituting the third “highgloss-polished” group (*n* = 10). The polishing time for each specimen was noted.

For the “intermediate-polish” group, one surface of each specimen was pre-polished using the grinding paper (Corund SC 150, Hager & Werken GmbH & Co. KG, Duisburg, Germany), followed by a grain size of 180 inserted in the laboratory micromotor with control box and straighthand piece. For the final polishing, the specimens were brushed (Abraso-Soft Acryl, bredent GmbH & Co. KG, Senden, Germany) on the brushing machine (Poliereinheit PE5, Degussa AG, Hanau, Germany) with the pumice slury (Steribim super, BEGO GmbH & Co KG; Bremen, Germany) and then with the polishing paste (Universal Polishing Paste, Ivoclar Vivadent AG; Schaan; Liechtenstein), using lathe bristle brush. Finally, in order to achieve a glossy shine, the specimens were polished using the soft cloth wheel (Polirapid, Dr. Montemerlo GmbH & Co KG; Singen; Germany) and paste (Universal Polishing Paste, Ivoclar Vivadent AG; Schaan, Liechtenstein). Finally, all polished specimens were cleaned in the ultrasonic bath (Stammopur RD5; Dr. H. Stamm GmbH; Berlin; Germany) for 1 min and left to dry.

### 2.3. Coating

In addition to the polishing procedure, twenty more additively produced specimens were thin coated with the same unpolymerized resin, which was used for the printing process, and cured in the light-curing unit (Otoflash G171, NK-Optik, Baierbrunn, Germany). The resin was added in a minimal layer thickness with a bristle brush until the visible staircase effect was filled. 10 of them were left in a coated state and constituted the “coated” group (*n* = 10) The 10 other coated specimens were additionally highgloss-polished using the protocol mentioned above and constituted the “coated + polished” group.

### 2.4. Surface Roughness

The surface roughness was measured for the “reference”, “intermediate”, “polished” and “coated” groups with a profilometer (Mahr SP6, Mahr GmbH, Goettingen, Germany). In order to ensure the reproducibility of all measurements, a position guide with a field of 10 × 10 mm was used to position all blocks on the same point. The measuring probe drove through with 15 single profiles with a point distance of 0.5 µm through the middle section (10 × 10 mm) of each specimen. The acquired data was analyzed in measuring software (MountainsMap universal 7.3, Digital Surf SARL, Besançon, France) and converted into profile curves. The average profile depth (R_a_) and maximal profile deviation (R_z_) were obtained for each specimen. Furthermore, the color map based on the R_z_ value was generated for one specimen from each group.

### 2.5. Gloss

The gloss value (gloss Units (GU)) was obtained with the Picogloss 560 MC (Erichsen GmbH & Co. KG; Hemer; Germany). The middle section of the specimens were placed in the measuring field. On each specimen, 15 repeated measurements were performed from different directions.

### 2.6. REM

For one specimen from each group, an area of 10 × 10 mm in the middle of the specimen was cut out with a cutting disc, sputter coated with gold-palladium (SCD005, Bal-Tec GmbH, Schalksmühle, Germany) and examined under a scanning electron microscope (LEO 1430; Zeiss; Oberkochen, Germany). The captions with 1000 magnification were obtained for the optical surface analysis.

### 2.7. Statistical Analysis

All gathered data was analyzed with JMP 13.1 software package (SAS Corp., Heidelberg, Germany). The measurements were tested for normality of distribution by goodness of fit with Shapiro-Wilk test using *p* < 0.05. As all data was distributed normally, the Tukey HSD test was applied on the α level of 0.05 The Pearson analysis was performed to reveal correlation between the polishing time and surface roughness.

## 3. Results

### 3.1. Surface Roughness

**Figure 3:**
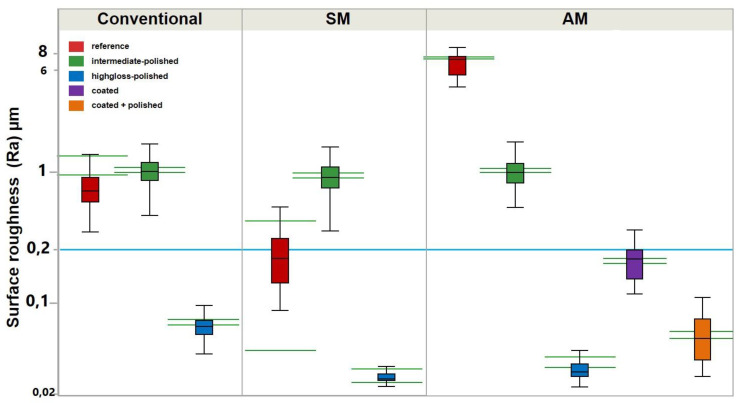
Surface roughness (R_a_) of auto-polymerizing (conventional), milled (SM), and 3D printed (AM) denture base materials. The blue line represents the threshold of clinical relevance (0.2 µm). The green horizontal lines represent the confidential intervals for each dataset.

**Figure 4:**
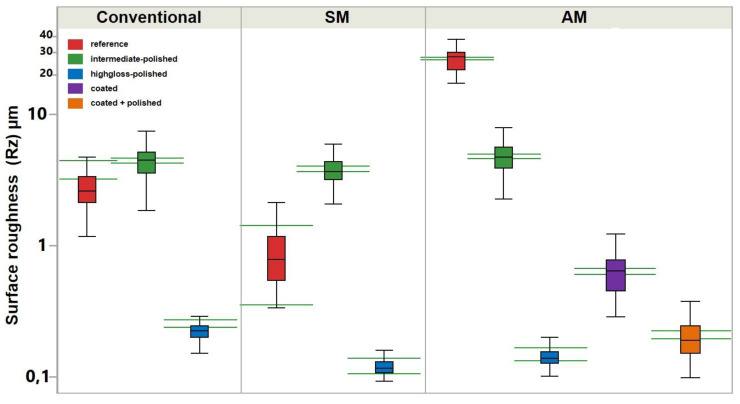
Surface roughness (R_z_) of auto-polymerizing (conventional), milled (SM), and 3D printed (AM) denture base materials. The green horizontal lines represent the confidential intervals for each dataset.

The profilometrical analysis revealed significantly higher R_a_ and R_z_ values in the AM group between all “reference” specimens (Figure 3 and Figure 4; Table 1). For “intermediate-polished” specimens, no statistical difference was observed between conventional, SM and AM groups. The “highgloss-polished” specimens showed statistically lower R_a_ and R_z_ values in the SM, followed by AM and conventional groups. Thus, the first hypothesis was rejected.

The analysis of R_a_ and R_z_ values in the AM group showed statistical difference between “highgloss -polished”, “coated + polished”, and “coated” specimens, respectively, meaning the second hypothesis must also be rejected.

The polishing time to achieve an optically gloss surface ranged between 5 min 40 s to 6 min 20 s. The Pearson analysis revealed no correlation between the polishing time and R_a_ and R_z_ values (*p* = 0.8), meaning that the difference of 20 s did not affect the final surface quality.

### 3.2. REM

**Figure 5:**
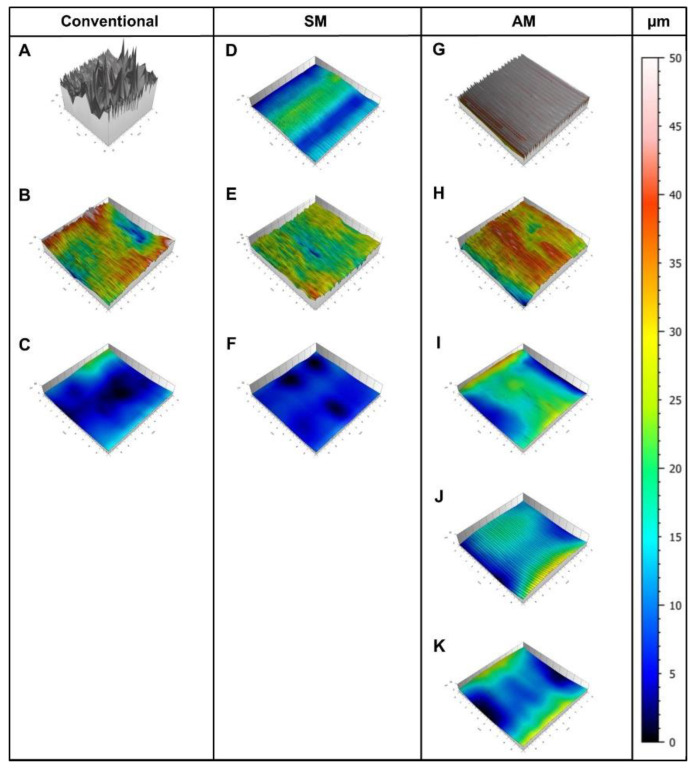
Profilometer scan images in conventional group (**A**): reference; (**B**): intermediate-polished; (**C**): highgloss-polished), SM group (**D**): reference; (**E**): intermediate-polished; (**F**): highgloss-polished), and AM group (**G**): reference; (**H**): intermediate-polished; (**I**): highgloss-polished; (**J**): coated; (**K**): coated + polished).

**Figure 6:**
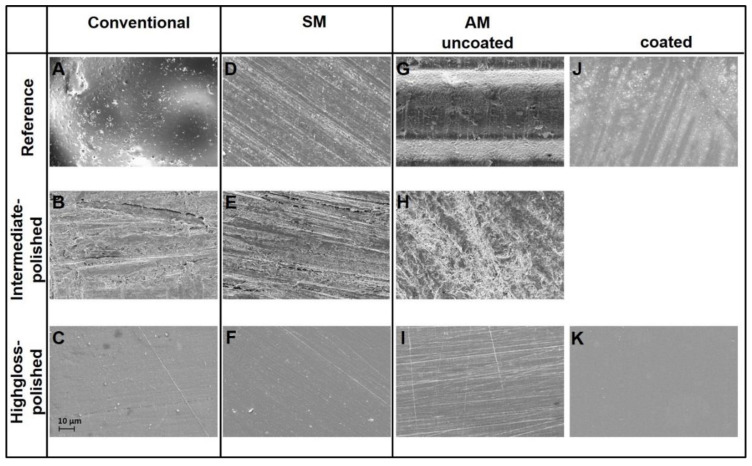
REM images in conventional group (**A**): reference; (**B**): intermediate-polished; (**C**): highgloss-polished), SM group (**D**): reference; (**E**): intermediate-polished; (**F**): highgloss-polished), and AM group (**G**): reference; (**H**): intermediate-polished; (**I**): highgloss-polished; (**J**): coated; (**K**): coated + polished).

The optical analysis of the REM pictures revealed substantial differences regarding the appearance of the specimens’ surface. The reference specimens in the conventional group showed a porous surface full of cavities, which were unevenly distributed over the whole specimens surface (Figure 5A). The reference specimens in the SM group demonstrated numerous parallel oriented lines, which were left by the milling cutter (Figure 5D and Figure 6D) In case of AM group, the 45° printed specimens demonstrated repeated oblique ridges, which refer to the layer-wise printing process (Figure 5G and Figure 6G).

The “intermediate-polished” specimens did not demonstrate any crucial differences in the surface topography and were characterized by combination of round, linear and amorphous shapes (Figure 6B,E,H).

The “highgloss-polished” specimens in all groups were generally similar to each other and were characterized by variously oriented patterns. The major difference between SM (Figure 6F) and AM groups (Figure 6I) touched upon the density of detected patterns.

The coating material covered the ridges on the printed (AM) specimens and results in relatively smooth surface full of small unevenly distributed patches and air bubbles. The most smooth and uniform surface was demonstrated by “coated + polished” specimens.

### 3.3. Gloss

**Figure 7:**
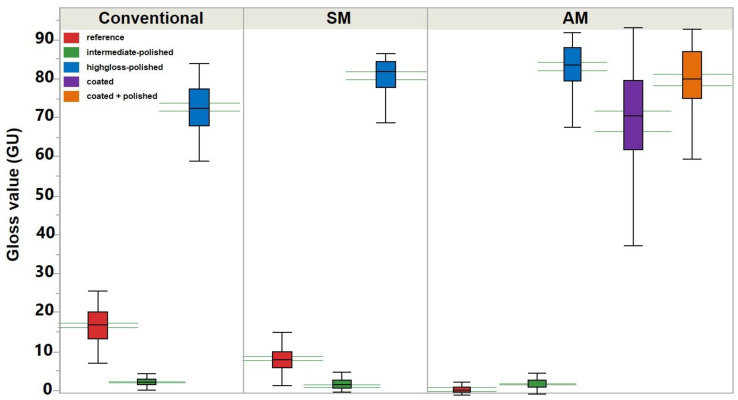
Gloss value of auto-polymerizing (conventional), milled (SM), and 3D printed (AM) denture base materials. The green horizontal lines represent the confidential intervals for each dataset.

In the polished specimens a significantly higher gloss value (GU) was revealed for AM, followed by SM and conventional groups (Figure 7) (Table 1). The intragroup analysis of AM revealed significantly higher gloss value for the polished specimens, followed by “coated + polished” and “coated” groups.

## 4. Discussion

The printing direction was reported to affect mechanical properties of printable resin materials [24]. This issue also applies to the surface characteristics of additively manufactured denture bases. Thus, the 45° printing direction may yield greater Ra values (up to 1.09 ± 0.07 µm), than 0° and 90°, as the stepwise layer orientation produces repeated oblique edges on the specimens surface [25]. In the present study all AM specimens were printed with 45° orientation and even greater R_a_ values of 6.61 ± 1.18 µm were encountered.

In opposite, the “reference” specimens showed even lower R_a_ values in the SM group than in conventional group. This may be attributed to numerous microscopic air bubbles and voids in the surface of auto-polymerizing resin.

In order to neglect this negative effect of the printing process and concentrate on the polishability of the used resin types as such, the “intermediate” group was formed and served as an equalizer for all testing groups. Thus, no statistically significant differences were observed in the “intermediate” pre-polished specimens. All three specimen groups were provided with the equal surface roughness, prior to be subjected to the final polishing.

After the final polishing significant differences in R_a_ and R_z_ values were observed for AM and SM compared to conventional group, ranging from 0.05 ± 0,02 to 0.02 ± 0 µm. These values lay below the threshold of clinical relevance, as described by Quirynen et al. and Bollen et al. [9,10]. Greater R_a_ values after the final polishing were reported for the subtractively manufactured specimens by Alp et al. (0.08 µm), Al-Dwairi et al. (from 0.12 ± 0.02 to 0.16 ± 0.03 µm), Srinivasan et al. (0.37 ± 0.03 µm) for various manufactures, which shows the anisotropic behavior of pre-polymerized PMMA [15,16,17].

In the present study, the encountered mean R_a_ values for polished specimens in the SM group were 0.02 ± 0 µm, using Vita pre-polymerized PMMA. In the topical literature only one study investigated the surface characteristics of this material in an untreated state and reported R_a_ and R_z_ values comparable with the present study (0.28 ± 0.07 µm and 1.26 ± 0.36 µm respectively) [26].

The highlighted differences in R_a_ values between the mentioned studies may also be attributed to the different polishing techniques and instruments used. Thus, as reported in the study of Kuhar et al. the auto-polymerized and heat-polymerized specimens, which were finally polished with the Universal Polishing Paste (Ivoclar Vivadent AG, Schaan, Liechtenstein)—as used in the present study—reached the R_a_ values from 0.04 ± 0 to 0.02 ± 0.01 µm, which coincide with results of the present study. The studies of Rao et al. and Gundor et al. confirm the statement that the polishing paste with soft cloth wheel yields a better surface quality allowing to achieve the in the R_a_ value in the order of 0.03 ± 0.06 µm [27,28].

In the present study the DLP method (Rapidshape D30II, Rapid Shape GmbH, Heimsheim, Germany) with a layer thickness of 50 µm was used. The resolution of the *z*-axis can have a great influence on the surface properties. This device is potentially capable of printing with a layer thickness of up to 25 µm. However, halving the thickness of the layer will double the printing time with reduced accuracy [29,30]. Therefore, a layer thickness of 50 µm was considered as a clinically acceptable compromise between surface quality, printing time and accuracy in dental applications. Future developments in printing hardware and software may possibly eliminate the “staircase” effect, resulting in a better surface quality of the denture bases, thus reducing the polishing time.

**Figure 8:**
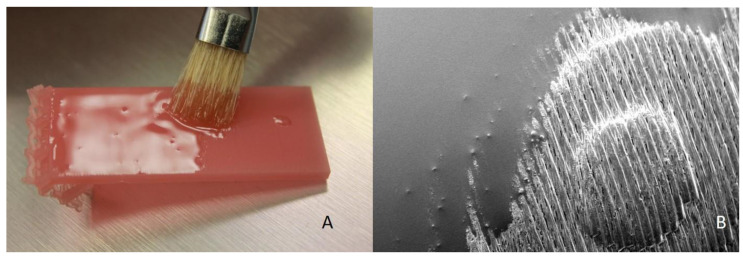
(**A**): Coating of the printed specimens with the unpolimerized resin from the 3D printer using a fine brush; (**B**): REM image of the coating application. The liquid unpolimerized resin flattens the staircase effect of the printed specimens.

Coating with various glazing materials was reported as an alternative to the traditional mechanical polishing. Choi et al. reported the R_a_ values from 0.26 ± 0.01 to 0.15 ± 0.02 µm, which means that some denture coating materials exceed the threshold of 0.2 µm and may compromise the clinical performance of a full denture they are applied on. In the present study one layer of the same denture resin material, which was used for the 3D printing of the specimens was applied with the brush onto the surface (Figure 8). Solely coated specimens demonstrated statistically greater R_a_ value of 0.16 ± 0.04 µm to the polished, but remained within the level of 0,2 µm. Coating in addition with polishing does not yield any significant superior outcome compared to the solo polishing.

For this reason the described way of coating may be proposed as an alternative to the conventionally polished specimens. Furthermore, in clinical and financial terms the application of such coating using the fluid denture resin reduced the production time and does not necessitate the purchase of any glazing materials, therefore allowing for a more efficient digital workflow.

In addition, it must be said that careful interpretation of the obtained R_a_ and R_z_ values is important, when analyzing the surface characteristics in clinical terms. For instance, even though the “reference” specimens in SM group showed R_a_ value of <0.2 µm (0.17 + 0.08 µm), it does not necessarily mean that an untreated subractively (SM) manufactured prosthesis could be applied by the patient. Although, it may be considered, whether the polishing process of milled prostheses can be reduced to a pure high-gloss polish, since treatment with sandpaper increases the roughness again. Furthermore, the mentioned studies of Quirynen et al. and Bollen et al. [9,10] dealt with titanium and were performed over 25 years ago. Thus, they may not faithfully represent the clinical situation, when using polymers for complete dentures and this fact calls for additional research to this topic.

The REM and profilometrical analysis revealed an optically uniform surface for both polished and coated specimens in all testing groups. However, the best characteristics were encountered in the REM image of the “polished + coated” specimens, which was completely devoid of any patterns. In the profilometer scan images a smooth surface was revealed for all polished and coated specimens, whereby the most uniform surface was observed by the polished SM specimens.

The polished specimens demonstrated the highest gloss value in the AM group. Significantly lower values were revealed for the coated specimens. The clinical interpretation of these differences may be assessed in further studies.

In the present study all specimens were evaluated right after production and no artificial aging was conducted. Alp et al. demonstrated the negative effect of the coffee thermocycling on the surface roughness of subtractively produced specimens [15]. It can be assumed that the time passage may negatively affect the coating material used in the present study. Thus, the influence of alternative artificial aging modalities of the surface characteristics of polished and coated additively produced denture resin should be evaluated in further studies.

In terms of coated denture surface, Choi et al. reported no difference in the surface roughness of five various glazing materials applied onto the heat-polymerized denture resin [5]. In the present study the additively produced specimens were coated with the same uncured resin, and its behavior after artificial aging must be investigated in future researches.

Although only one experienced operator was used for polishing to avoid variation in technique and pressure, using samples imitating the surface size of a full denture, the study may be repeated with a machine controlled polish and ISO normative measurements to avoid individual variations.

## 5. Conclusions

Within the limitations of the present in-vitro study, the following conclusions can be made:

1. The milled specimens demonstrated superiors surface characteristics than 3D printed and conventionally produced after polishing, whereby all groups showed the Ra values within the clinically relevant threshold of 0.2 µm.
2. The polished specimens demonstrated superior surface characteristics compared to coated specimens, whereby all groups showed the Ra values within the clinically relevant threshold of 0.2 µm.
3. The polished specimens showed superior gloss value in the AM group, followed by SM and conventional.
4. The coating of additively produced dentures with the same unpolymerized material may be regarded as an alternative to conventional polishing.

## Figures and Tables

**Table 1:** Mean and SD values for “reference”, “intermediate-polished”, “highgloss-polished”, “coated”, and “coated + polished” specimens in conventional, SM, and AM groups. The letters *a*, *b*, and *c* indicate the *p*-value according to the Tukey HSD test.

		Conventional	SM	AM
		Mean ± SD	Mean ± SD	Mean ± SD
**Ra (µm)**	**Reference**	0.83 ± 0.92 *^a^*	0.17 ± 0.08 *^b^*	6.61 ± 1.18 *^c^*
	**Intermediate-polished**	0.76 ± 0.19 ^*a*^	0.69 ± 0.19 ^*a*^	0.84 ± 0.2 ^*a*^
	**Highgloss-polished**	0.05 ± 0.02 ^*a*^	0.02 ± 0 ^*b*^	0.03 ± 0.01 ^*c*^
	**Coated**			0.16 ± 0.04 ^*c*^
	**Coated + polished**			0.04 ± 0.02 ^*c*^
**Rz (µm)**	**Reference**	3.61 ± 3.15 ^*a*^	0.95 ± 0.38 ^*b*^	27.64 ± 4.76 ^*c*^
	**Intermediate-polished**	4.13 ± 1 ^*a*^	3.63 ± 0.91 ^*a*^	4.44 ± 1.12 ^*b*^
	**Highgloss-polished**	0.31 ± 0.2 ^*a*^	0.16 ± 0.03 ^*b*^	0.19 ± 0.05 ^*c*^
	**Coated**			0.73 ± 0.22 ^*c*^
	**Coated + polished**			0.26 ± 0.1 ^*c*^
**Gloss value** **(GU)**	**Reference**	16.93 ± 4.29 ^*a*^	8.69 ± 3.19 ^*b*^	1.10 ± 0.82 ^*c*^
	**Intermediate-polished**	2.77 ± 0.6 ^*a*^	2.37 ± 0.82 ^*a*^	1.77 ± 0.66 ^*a*^
	**Highgloss-polished**	70.78 ± 5.81 ^*a*^	78.55 ± 3.93 ^*b*^	80.84 ± 7.94 ^*c*^
	**Coated**			67.25 ± 13.65 ^*c*^
	**Coated + polished**			77.39 ± 7.84 ^*c*^
